# Comprehensive analysis of LRR-RLKs and key gene identification in *Pinus massoniana* resistant to pine wood nematode

**DOI:** 10.3389/fpls.2022.1043261

**Published:** 2022-12-14

**Authors:** Ziyan Nie, Wenhua Li, Lili Deng, Kai Gao, Qinghua Liu, Zhichun Zhou

**Affiliations:** ^1^ Research Institute of Subtropical Forestry, Chinese Academy of Forestry, Engineering Research Center of Masson Pine of State Forestry and Grassland Administration, Key Laboratory of Tree Breeding of Zhejiang Province, Hangzhou, Zhejiang, China; ^2^ Nanjing Forestry University, Nanjing, China

**Keywords:** *pinus massoniana*, receptor-like kinase, LRR-RLKs, expression pattern, PTI

## Abstract

*Pinus massoniana* is a pioneer tree widely planted for afforestation on barren hills in southern China where the total planted area is 8.04 million ha. The invasive pine wood nematode (*Bursaphelenchus xylophilus*) poses a serious threat to the survival of *P. massoniana*. Plant resistance genes encoded by leucine-rich repeat-containing transmembrane-receptor proteins play important roles in plant defense. Leucine-rich repeat receptor-like kinases (LRR-RLKs), the largest subfamily of the RLK protein family, play an important role in sensing stress signals in plants. However, the LRR-RLKs of *P. massoniana* have not been characterized previously, and their role in resistance to *B. xylophilus* is unknown. In this study, 185 members of the LRR-RLK subfamily were identified in *P. massoniana* and were categorized into 14 subgroups. Transcriptomic and quantitative real-time RT-PCR analyses showed that *PmRLKs32* was highly expressed in the stem tissue after inoculation with *B. xylophilus*. The gene exhibited high homology with *AtFLS2* of *Arabidopsis thaliana*. *PmRLKs32* was localized to the plasma membrane and was significantly upregulated in nematode-resistant and nematode-susceptible individuals. The transient expression of *PmRLKs32* resulted in a burst of reactive oxygen species production in *P. massoniana* and *Nicotiana benthamiana* seedlings. These results lay a foundation for further exploration of the regulatory mechanism of LRR-RLKs in response to biotic stress in *P. massoniana*.

## Introduction

Plants are exposed to diverse biotic stresses in nature. A plant must respond to adverse biotic factors for survival and to maintain growth. Pests are a biotic stress that seriously threaten the growth and development of trees. Plants rely on their defense system, which predominantly includes pattern-triggered immunity (PTI) and effector-triggered immunity (ETI), to resist pathogen attack (Naveed et al., 2020). Recent studies have shown that PTI and ETI synergistically enhance disease resistance in plants, and the disease response pathways of each system show a certain degree of overlap. The ETI response activates the MAPK signaling pathway and the production of reactive oxygen species (ROS) (Yuan et al., 2021b). The activation of ETI requires the participation of pattern-recognition receptors (PRRs) and co-receptors. The ETI signal can mobilize the PTI pathway and, in turn, amplify the PTI response (Ngou et al., 2021; Pruitt et al., 2021; Tian et al., 2021; Yuan et al., 2021a). In addition, the concept of the “resistosome” has been proposed (Bi et al., 2021). These new views and concepts indicate that research in the field of plant immunity is entering a new phase and lay a crucial theoretical foundation for targeted improvement of the plant immune system.

The PTI pathway involves recognition of conserved pathogen-associated molecular patterns (PAMPs), microbe-associated molecular patterns (MAMPs), and damage-associated molecular patterns (DAMPs) by PRRs (Abdul Malik et al., 2020). The PRRs, which mainly comprise receptor-like kinases (RLKs) and receptor-like proteins (RLPs), play an important role in the first-tier basal immunity of plants that is triggered by the recognition of external pathogens (Lin et al., 2020). The PRRs elicit downstream cellular responses, including defense gene expression, ROS production, and callose deposition, by recognizing highly conserved molecular structures or characteristics of pathogens and pests (Albert et al., 2019). However, the recognition mechanism and the cell surface and intracellular synergistic mechanisms remain unclear.

The leucine-rich repeat receptor-like kinase (LRR-RLK) subfamily, the largest group within the plant RLK family, is mediated by many cellular signal transduction pathways. The proteins of this family contain three functional domains. The extracellular domain (ECD) perceives signals that contain varying numbers of LRR repeats. The transmembrane domain connects the internal and external cellular compartments. The intracellular kinase domain (KD) can transmit a signal through phosphorylation (Chakraborty et al., 2019)—for example, *Arabidopsis thaliana FLAGELLIN-SENSITIVE 2* (*AtFLS2*) senses the flagellin elicitor flg22 to activate a relatively low-level defense response, and *EF-TU RECEPTOR* (*AtEFR*) mediates plant resistance to a bacterial pathogen. *RECEPTOR-LIKE KINASE 902* (*AtRLK902*) may directly bind to and phosphorylate the downstream gene *BRASSINOSTEROID-SIGNALING KINASE 1* (*BSK1*), thereby transmitting an immune signal downward (Zhao et al., 2019). *OsLRR-RLK1* is involved in the regulation of pest defense responses in rice (Ye et al., 2019). Thus, the LRR-RLK family plays important roles in responses to abiotic and biotic stresses.

*Pinus massoniana* is the main timber species grown in southern China and is adaptable to diverse topography and environments. The pine wood nematode (*Bursaphelenchus xylophilus*) feeds on cells in the vascular bundles of the tree. The resulting pine wilt disease is devastating and threatens the survival of *P. massoniana*. In previous studies, we identified several *P. massoniana* accessions that showed high resistance or susceptibility to *B. xylophilus*, from which we selected representative resistant and susceptible individuals for full-length transcriptome sequencing. Through transcriptomic analysis of gene families of *P. massoniana*, in this study we identified candidate LRR-RLK genes and selected the most representative gene for preliminary functional verification. The results provide a foundation for the future studies of pine wood nematode resistance.

## Materials and methods

### Identification of LRR-RLK family members

Transcriptome data for *P. massoniana* were derived from the previously reported transcriptome of resistant and susceptible individuals post-inoculation with *B. xylophilus* (Liu et al., 2017). TBtools software was used to extract all LRR-RLK genes from the transcriptome that contained the LRR domain (PF13855). The default parameters were used for screening, and the E-value was set to E < 10−20. HMMER (https://www.ebi.ac.uk/Tools/hmmer/results/032E22B6-BB2E-11EC-94AB-5DE9DBC3747A/score) and CD-Search (https://www.ncbi.nlm.nih.gov/cdd/) were used to screen protein sequences of *P. massoniana* containing the complete LRR domain and beginning with a Met residue. A Perl script in the Linux system was used to delete the duplicated sequences among the selected *P. massoniana* resistance protein sequences. Based on previous reports and functional annotations of *A. thaliana* LRR-RLK proteins, the LRR-RLK proteins that included the LRR and protein kinase motifs were downloaded from Phytozome v13 (https://phytozome-next.jgi.doe.gov/) (Xi et al., 2019). The selected LRR-RLK proteins were further screened with CD-Search.

### Sequence analysis and phylogenetic analysis

TBtools software was used to predict the molecular weight and isoelectric point of the LRR-RLK proteins in batches. CELLO (http://cello.life.nctu.edu.tw/) and PSORT (https://psort.hgc.jp/) were used for subcellular localization prediction (Chen et al., 2020). An evolutionary tree was reconstructed using the maximum-likelihood method based on a multiple sequence alignment of the LRR-RLK protein sequences of *P. massoniana* and *A. thaliana* with MEGA software. Support for the topology of the phylogeny was assessed by performing a bootstrap analysis with 1,000 replicates. The Multiple Expectation Maximization for Motif Elicitation (MEME) tool (http://meme-suite.org/tools/meme) was used to analyze the conserved motifs of the LRR-RLK proteins with the following parameters: minimum and maximum motif widths of six and 50, respectively, and maximum pattern number 10. The fragments per kilobase of exon per million mapped fragment values of the *P. massoniana* transcripts were used in conjunction with the read map per thousand bases exon model per million values for the nematode to estimate the abundance of *P. massoniana* LRR-RLK gene transcripts. The relative expression level of the control was set to “1”.

### Plant material and treatments

Two-year-old *P. massoniana* seedlings were obtained from the Linhai forest farm (28°40′–29°04′ N, 120°49′–121°41′ E), Linhai Province, China. To study the expression level of LRR-RLK genes, we selected three seedlings of uniform growth for nematode treatment. Each plant was inoculated with 1,000 heads. Samples of stem tissue were collected at 0, 1, 15, and 30 days after inoculation.

### RNA extraction and quantitative real-time reverse transcription PCR

Total RNA was extracted from samples using the EASYspin Plus Complex Plant RNA Kit (RN53, Aidlab, Beijing, China). The RNA concentration and purity were measured with a NanoDrop 2000 spectrophotometer (Thermo Fisher Scientific, Waltham, MA, USA), and the RNA integrity was estimated by agarose gel electrophoresis. The first-strand cDNA was synthesized using the HiScript III All-in-one RT SuperMix Perfect for qPCR (R333, Vazyme, Hangzhou, China). Primers for quantitative real-time reverse transcription PCR (qRT-PCR) were designed using Primer 5.0 (**Supplementary Table S1**). The Taq Pro Universal SYBR qPCR Master Mix (Q712, Vazyme) was used to amplify the target sequence. Each PCR mixture (20 µl) contained 2 µl diluted cDNA (10× dilution), 10 µl SYBR Green Real-time PCR Master Mix, 0.4 µl of each primer (10 µM), and 7.2 µl ddH_2_O. The RT-PCR program accorded with the kit manufacturer’s instructions. The β-tubulin gene was used as an internal reference, and three biological replicates and three technical replicates were analyzed for each sample. Information on the primers used is presented in **Supplementary Table S1**. The relative expression levels were calculated using the 2^−ΔΔCt^ method. The IBM SPSS Statistics for Windows, version 19.0 (IBM Corp, Armonk, NY, USA), was used to estimate the significance of differences between means with Student’s *t*-test (*P < 0.05, **P < 0.01).

### Subcellular localization assay

The construct 35S:GFP-*PmRLKs32* was generated by homologous recombination. The 35S:GFP-*PmRLKs32* construct containing the green fluorescent protein (GFP) was transiently transformed into the leaves of *Nicotiana benthamiana* following a previously described method (Liu et al., 2017). The fluorescence signals were observed with a laser scanning microscope (LSM880, ZEISS, Jena, Germany).

### Transient transformation

Four-week-old *N. benthamiana* seedlings and young *P. massoniana* tissue-cultured seedlings were used as materials for transient transformation. We used the agroinfiltration method for transient expression of GFP-fused proteins in *N. benthamiana* leaves, and 35S:GFP was used as the control. First, 3-ml LB cultures supplemented with rifampicin and kanamycin in a capped 10-ml conical flask were inoculated with the plasmid. The cultures were incubated at 28°C under agitation (200 rpm) for 4 h. In the second step, the cells were pelleted by centrifugation for 15 min at 5,000 rpm. After aspiration of the supernatant, the pellet was resuspended in 3 ml of infiltration medium (10 mM MES, 10 mM MgCl_2_, and 200 μM acetosyringone). The samples were diluted to OD600 = 1 with the infiltration buffer. After letting it sit for 1 h, a 1-ml needleless syringe was filled with the suspension and injected into *N. benthamiana* leaves, which were incubated for 48 h in the dark. For *P. massoniana*, the suspension samples were introduced into the plant by vacuum infiltration. After incubation for 2 days, nitro blue tetrazolium (NBT) staining was performed, and RNA was also extracted from the plants for quantitative analysis.

## Results

### Verification of LRR-RLK genes in *P. massoniana*

A total of 185 candidate genes corresponding to LRR-RLK genes were screened from the transcriptome data of *B. xylophilus*. The statistics for these LRR-RLK genes are shown in **Supplementary Table S2**. The number of amino acid residues predicted to be encoded by these genes ranged from 470 to 1,460. The LRR-RLK proteins comprising more than 900 amino acids were the most abundant, whereas 54 LRR-RLK proteins comprised fewer than 900 amino acids. The relative molecular weights of the LRR-RLK proteins were 52–160 kDa. Prediction of the subcellular localization suggested that the LRR-RLK proteins were widely distributed within the cell: 112 LRR-RLK proteins were predicted to be mainly localized in the plasma membrane, 14 mainly in the nucleus, 18 in the vacuole, 10 in the extracellular matrix, three in the endoplasmic reticulum, four in the cytoplasm, and 24 predominantly in the chloroplasts. Based on these observations, we identified the LRR-RLK genes that encoded proteins mainly localized to the plasma membrane and selected for further analysis. Other predicted physical and chemical properties of the LRR-RLK proteins are shown in **Supplementary Table S2**.

### Motifs and phylogenetic analysis of LRR-RLK proteins

**Figure 1:**
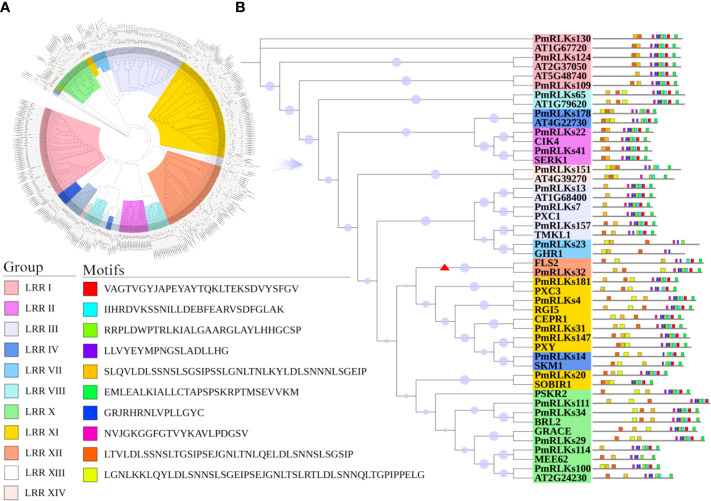
Motifs and phylogenetic tree for PmRLK proteins. **(A)** Phylogenetic tree based on the full-length amino acid sequences using the maximum likelihood method. The different subgroups are indicated by different colors. **(B)** Motifs and phylogenetic tree for *P. massoniana and A. thaliana* homologs. The red arrowhead shows the genes that were selected for further analysis.

Phylogenetic analysis revealed that the 185 LRR-RLK proteins of *P. massoniana* could be divided into 14 groups consistent with the LRR-RLK protein classification for *A. thaliana*. The conserved shared motifs were determined using the full-length amino acid sequences of the LRR-RLK proteins with the MEME tool. Ten motifs were identified in the 185 LRR-RLK protein sequences of *P. massoniana* ranging in length from 16 to 50 amino acids. The genes of *A. thaliana* and *P. massoniana* placed in the same lineage were summarized, and a motif structure map was generated. Motif 1 was the main feature (**Figure 1**). The functions of most of the motifs remain to be elucidated. In combination with the results of the phylogenetic analysis, the conservative composition of LRR domains strongly supported the reliability of the classification of the proteins into groups. Certain genes of the RLK subgroup had a higher homology with genes of *A. thaliana*, but the number of genes differed significantly among the subgroups, which may be the result of functional evolution. The number of *P. massoniana* genes was more than six times that of *A. thaliana* genes in subgroups of the LRR XII group (**Table 1**). This may be due to environmental selection that has resulted in gene duplication in *P. massoniana*, and these genes may play an important role in disease resistance pathways. Importantly, *PmRLKs32* was classified in the LRR XII group; this protein is induced by *B. xylophilus* and affects plant secondary metabolism (Liu et al., 2021).

**Table 1:** Number of genes in the subgroups of LRR-RLK genes from *Pinus massoniana* and *Arabidopsis thaliana*.

	Number of genes	
Subgroup	*P. massoniana*	*A. thaliana*
LRR I	20	46
LRR II	8	14
LRR III	15	44
LRR IV	1	3
LRR V	4	9
LRR VI	7	11
LRR VII	5	9
LRR VII	7	21
LRR IX	5	4
LRR X	12	16
LRR XI	46	41
LRR XII	51	8
LRR XIII	2	0
LRR XIV	2	2

### Transcriptome analysis of *P. massoniana*

**Figure 2:**
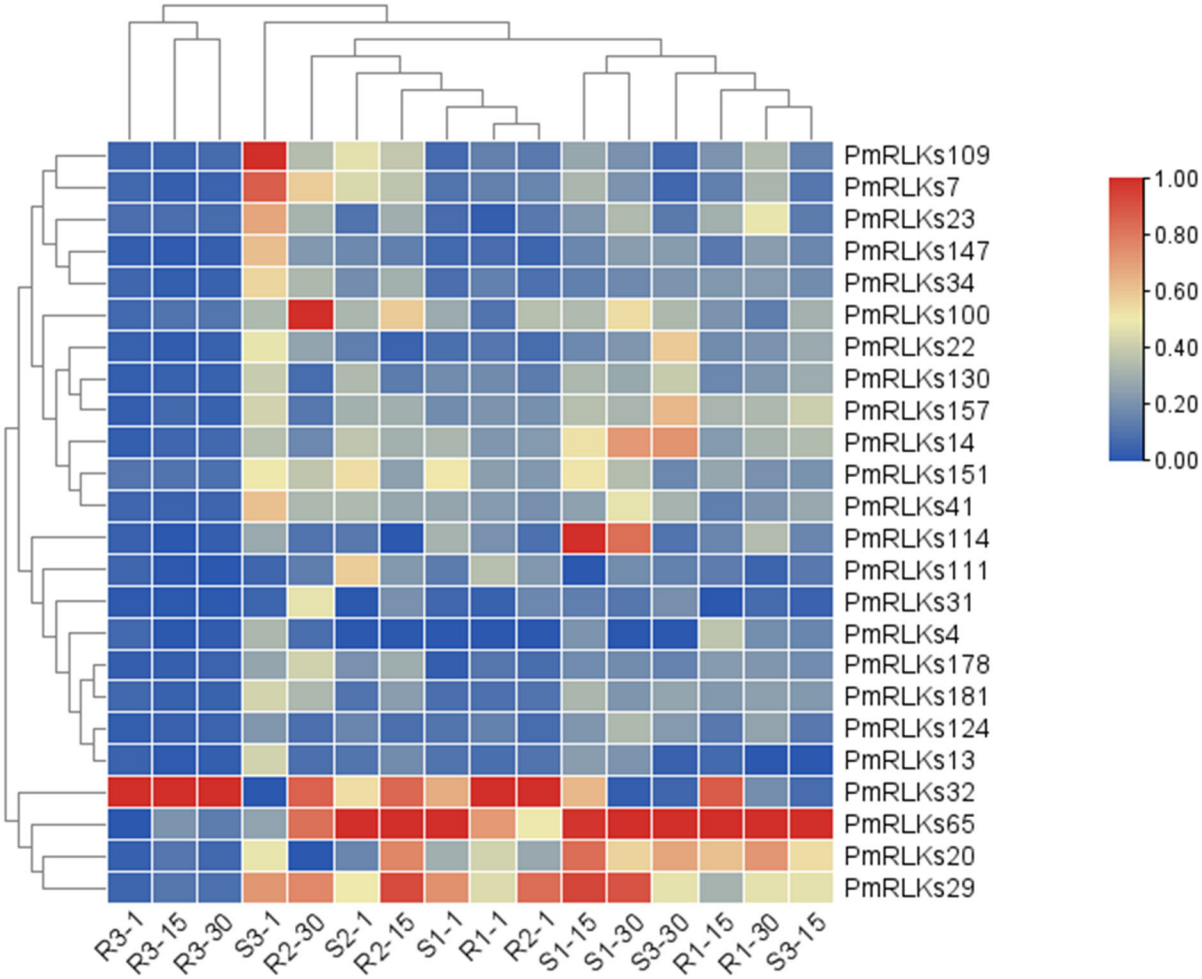
Heat map of PmRLK proteins. The PmRLK family members in *Pinus massoniana* that had high homology with the proteins of *Arabidopsis thaliana* are shown, and the heat map includes data for samples collected at 1, 15, and 30 days after inoculation. The expression level of the sample is relative to that of the control. The color scale represents the relative fold change in expression level.

The sequence information and heat map data for this analysis were based on the transcriptome. Combining the heat map (**Figure 2**) results and weighted gene co-expression network analysis of the transcriptome, 22 candidate *PmRLK* genes were identified (**Supplementary Figure S1**).

**Figure 3:**
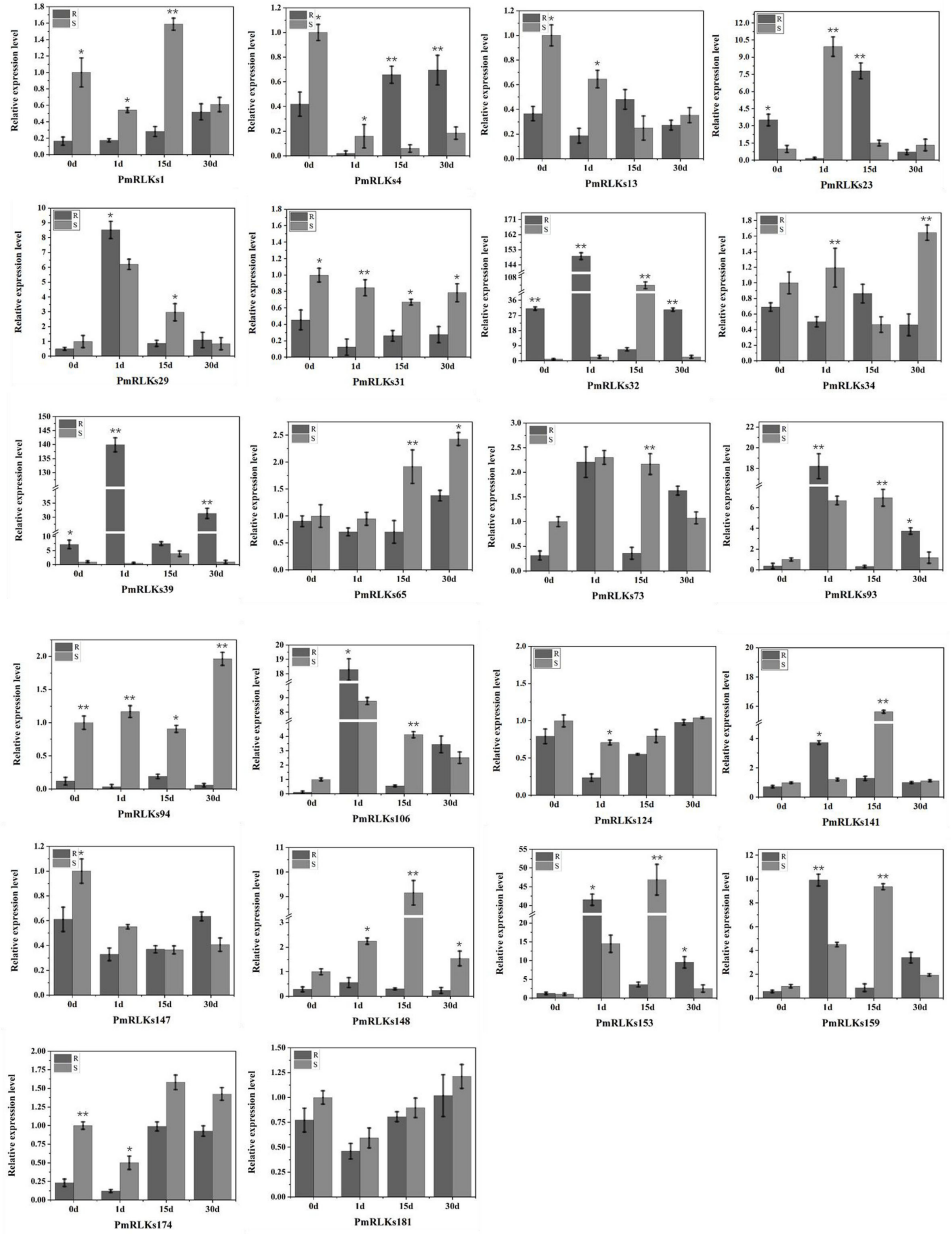
Relative expression of *PmRLK* genes of *P. massoniana* in response to nematode treatment. The relative expression levels are the log_2_ (2^−ΔΔ^ *^C^* ^t^) values. The asterisks indicate significant differences between each time point of highly resistant (R) and susceptible (S) accessions of *P. massoniana*. *p < 0.05, **p < 0.01.

A qRT-PCR analysis was conducted to determine the expression levels of the *PmRLK* genes. Consistent with the transcriptome data, the expression levels of *PmRLKs32* were significantly upregulated after infection. In the highly resistant sample, *PmRLKs32* was significantly upregulated at 1 day after inoculation, and the relative expression level exceeded the highest level observed for susceptible samples. On day 15 after inoculation, the *PmRLKs32* expression level decreased to less than the original level, and after 30 days it recovered to the original level. *PmRLKs39* was significantly upregulated at 1 day after inoculation. The trends for *PmRLKs153* and *PmRLKs159* were consistent with that for *PmRLKs32*, but the relative expression levels of the former two genes were slightly lower. The trend for *PmRLKs23* was opposite to that of *PmRLKs32*. Other *PmRLK* genes did not show a significant variation in relative expression levels in response to inoculation (**Figure 3**). The qRT-PCR results indicated that the samples with high resistance responded quickly to inoculation and maintained internal homeostasis. The *PmRLK* expression levels at 15 days post-inoculation were lower than those prior to inoculation, which may be due to negative feedback of plant immunity to prevent an excessive immune response from causing damage to the plant itself.

### Analysis of subcellular localization of *PmRLKs32*

**Figure 4:**
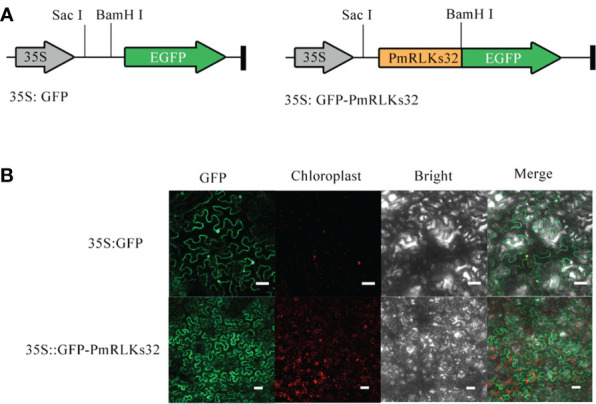
Subcellular localization of *PmRLKs32* in *Nicotiana benthamiana* leaf cells. **(A)** Schematic diagrams of the 35S:GFP vector and 35S:GFP-*PmRLKs32* construct. **(B)** Fluorescence from 5S:GFP and 35S:GFP-*PmRLKs32* transiently expressed in 4-week-old *N. benthamiana* leaves. The GFP signals were observed with a ZEISS LSM880 confocal microscope. Bar, 50 μm.

The *PmRLK* genes mainly participate in signal transduction. The predictions for their subcellular localization showed that they mainly play a role in the plasma membrane. To verify the prediction, *PmRLKs32* was selected for a subcellular localization experiment because it showed the highest relative expression level (**Figure 4**). Consistent with the prediction, *PmRLKs32* was localized to the plasma membrane. The PmRLKs32 protein may bind to extracellular effectors to activate immunity and stimulate the synthesis of relevant hormones to resist various stresses.

### Transient transformation with *PmRLKs32*

**Figure 5:**
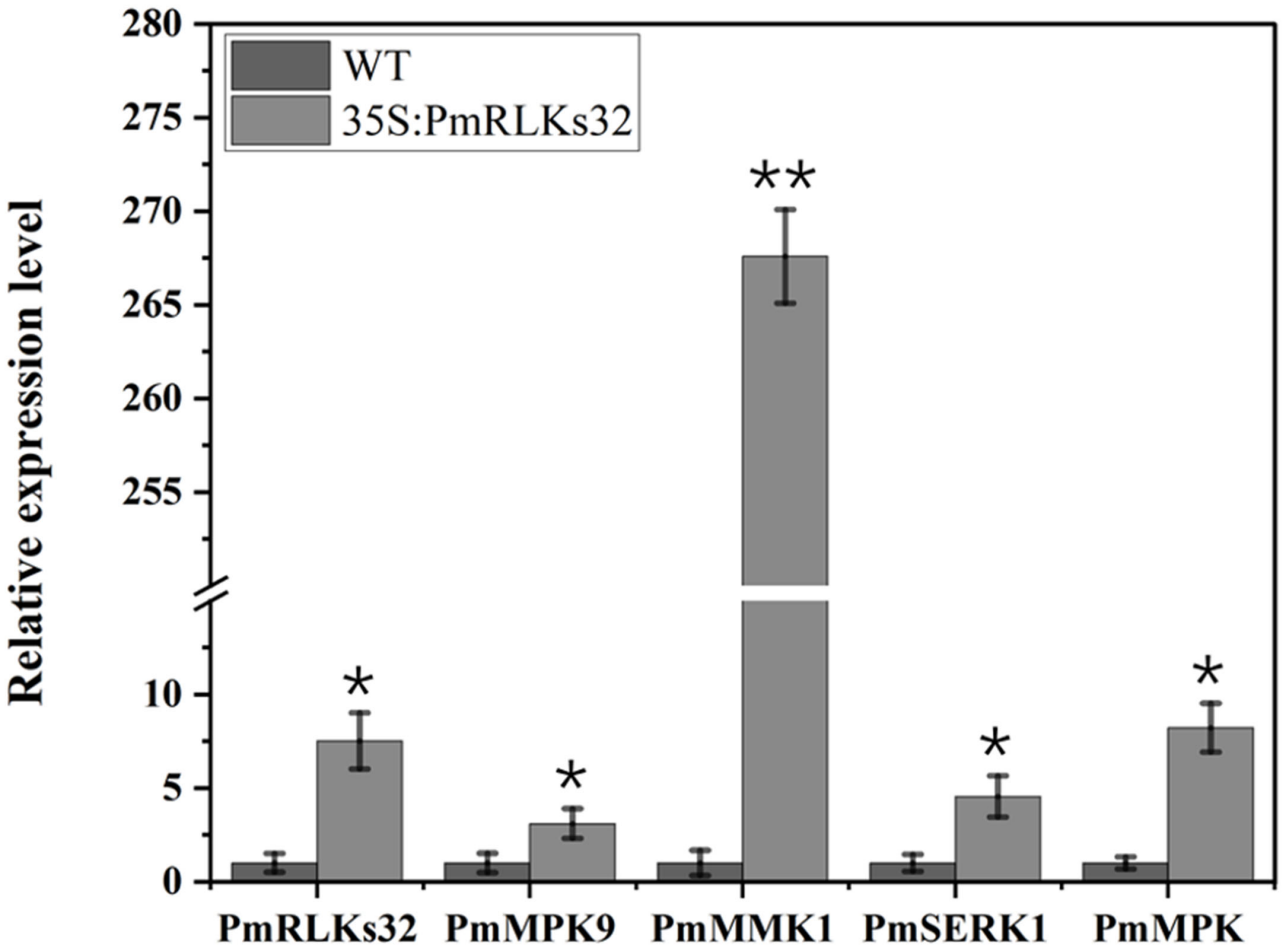
Gene expression downstream of *PmRLKs32*. The scale represents relative expression levels based on the log_2_ (2^−ΔΔ^ *^C^* ^t^) values. The asterisks indicate significant differences between each time point of the wild-type (WT) and 35S:*PmRLKs32*-overexpression plants. *p < 0.05, **p < 0.01.

**Figure 6:**
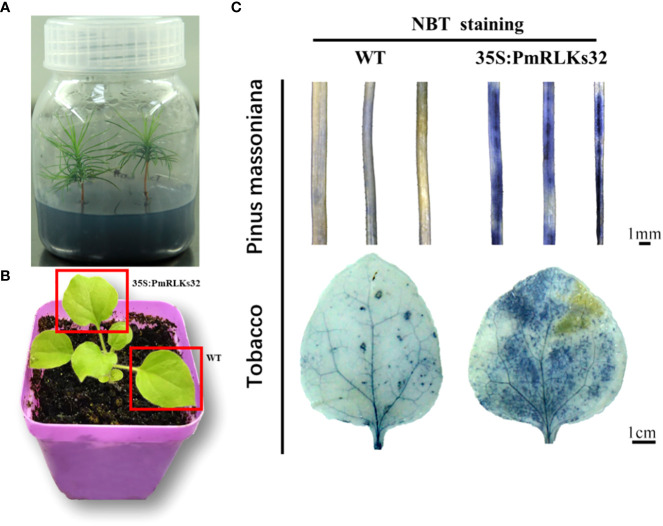
Staining of reactive oxygen species with nitro blue tetrazolium (NBT). **(A)** Tissue-cultured seedlings of *Pinus massoniana*. **(B)** Material of *Nicotiana benthamiana*: leaves used for transient transformation with the 35S:*PmRLKs32* construct are indicated by red boxes. **(C)** NBT staining of needles of *P. massoniana* (upper) and leaves of *N. benthamiana* (lower).

To further clarify the function of *PmRLKs32*, a transient transformation assay with *N. benthamiana* and *P. massoniana* was performed. The function of *PmRLKs32* was confirmed by qRT-PCR and histochemical visualization of ROS accumulation. The qRT-PCR results showed that *PmRLKs32* was successfully expressed in *N. benthamiana* and *P. massoniana*. The expression levels of downstream related genes and resistance-related transcription factors were detected in the transient overexpression plants. The results revealed that the expression of downstream genes of the PTI resistance pathway was upregulated, whereas the expression of ETI resistance pathway-related transcription factors was downregulated in the transient overexpression plants (**Figure 5**). In addition, NBT staining indicated that ROS and callose were significantly accumulated in the overexpression plants compared with those of the control. These results confirmed that *PmRLKs32* could activate the PTI pathway and enhance plant resistance through ROS production (**Figure 6**).

## Discussion

With plant evolution from unicellular eukaryotic algae to multicellular angiosperms, the number of RLK family members increased rapidly. In the present phylogenetic analysis, few genes showed a high homology between *P. massoniana* and *A. thaliana*, and the majority were phylogenetically well separated. The present results indicated that the *PmRLK* genes of *P. massoniana* may have undergone segmental duplication, tandem duplication, or polyploidization to adapt to various stresses in the environment. The tomato RLK proteins SlSERK3A and SlSERK3B enhance root-knot nematode resistance and interact with FLS2 *in vivo* (Peng and Kaloshian, 2014). FLS2 homologous genes have been reported in tobacco, *A. thaliana*, and tomato (Chinchilla et al., 2006; Hao et al., 2016; Zhou and Zeng, 2018). We identified the *PmRLKs32* protein in *P. massoniana*, which had a high homology to *AtFLS2* and was classified in the subgroup LRR XII, and demonstrated that overexpression of *PmRLKs32* triggered a burst of ROS production. As a consequence of stress metabolism, ROS not only play a role in plant defense mechanisms but also participate in biotic stress responses as signal molecules (Tarfeen et al., 2022). After the transient overexpression of *PmRLKs32* in *P. massoniana* tissue-cultured seedlings, the downstream genes *MPK9*, *MPK*, *MMK1*, and *SERK1* were also up-regulated. Therefore, overexpression of *PmRLKs32* stimulated the immune response in *P. massoniana*, but the specific mechanism is still unclear and requires further investigation.

Some redundant and truncated RLK genes currently without functional annotations are often ignored. In the phylogenetic tree, the number of *P. massoniana* genes in subgroup LRR XII exceeded the number of *A. thaliana* genes. The functions of these extra *P. massoniana* genes in subgroup LRR XII are currently unknown, and the proteins may act independently or as accessory proteins. In *A. thaliana*, *AtVRLK1* redundantly regulates secondary cell wall thickening (Huang et al., 2018). An additional family of resistance proteins, LRR-RLPs, may also play an indispensable role. The difference between RLK and RLP proteins is only in the C-terminus, and RLP proteins may even be truncated variants of RLK proteins (He et al., 2018). These RLP genes may have acquired novel functions as a result of the structural changes. The *RLP2* and *RLP3* genes only lack a C-terminal kinase domain compared with LRR-RLK genes containing the sulfate tyrosine 1 receptor (*PSY1R*) (Oehlenschlæger et al., 2017). The LRR-RLP gene *CLAVATA 2* has the same function as the LRR-RLK gene *CLAVATA 1* (Hanemian et al., 2016; Pan et al., 2016).

Comprehensive identification of *P. massoniana* LRR-RLKs and resolution of their phylogenetic relationships will facilitate the further exploration of genes with potentially redundant functions. The PTI and ETI pathways are the major defense pathways in plants. The diversity of genes is closely associated with the ability to adapt to stress. The present analysis revealed the potential function of *PmRLKs32* in resistance to pine wilt disease.

## Conclusions

This study comprehensively analyzed the RLK genes of *P. massoniana* and identified 185 *PmRLK* genes classified into 14 groups. Previous studies have shown that *RLK* genes play an important role in the plant defense system. A phylogenetic analysis revealed that *PmRLKs32* showed a high homology to *AtFLS2* of *A. thaliana*. This gene may play a crucial role in the defense regulatory network in *P. massoniana*. By performing qRT-PCR assays and transcriptome analysis, we confirmed that *PmRLKs32* was highly expressed in pine wood nematode-resistant individuals in response to nematode inoculation. Transient transformation with *PmRLKs32* revealed the accumulation of callose and ROS in the needles of *P. massoniana*. Hydrogen peroxide may activate the jasmonate pathway as a signal molecule, thereby enhancing plant resistance. The results further implicate *PmRLKs32* involvement in the defense regulatory network of plants. In addition, the present findings provide new insights into the function of *RLK* genes and provide a basis for resistance breeding and further study of the defense regulatory network in *P. massoniana*.

## Data availability statement

The original contributions presented in the study are included in the article/**Supplementary Material**. Further inquiries can be directed to the corresponding authors.

## Author contributions

ZN: Conceptualization, methodology, software, experiment, investigation, data analysis, and writing—original draft; WL: data curation and writing—original draft; LD: experiment and data analysis; KG: visualization, writing—review and editing, resources, and supervision; QL: software and validation; ZZ: funding acquisition and supervision. All authors contributed to the article and approved the submitted version.
