# Supplementary figures and images for: Comprehensive analysis of LRR-RLKs and key gene identification in Pinus massoniana resistant to pine wood nematode
Source: Front Plant Sci. 2022 Dec 14;13:1043261. doi: 10.3389/fpls.2022.1043261 (PMC9795191; doi:10.3389/fpls.2022.1043261)

**Figure S1. The heat map of PmRLKs proteins.**

**
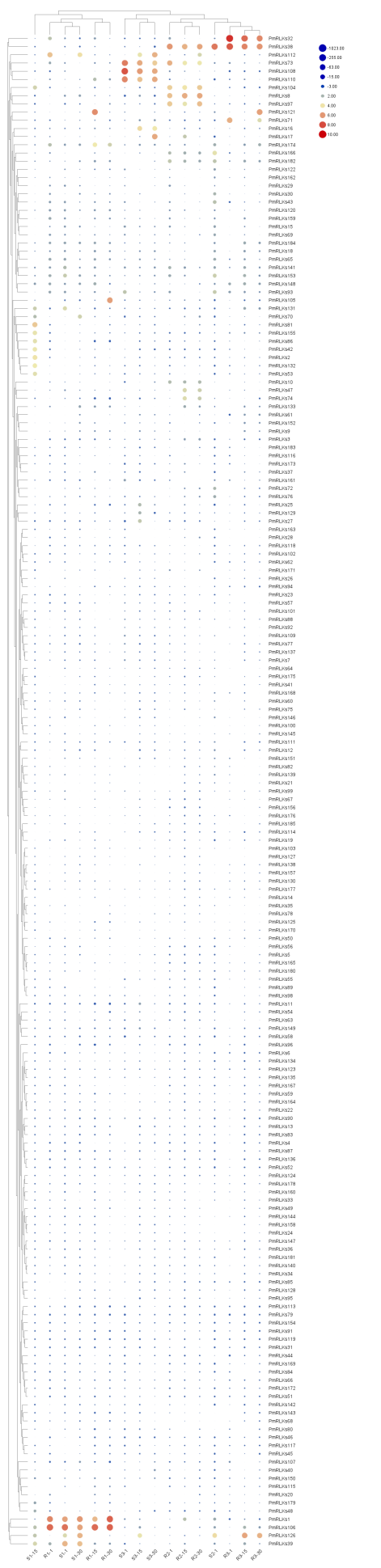
**

Supplement: Supplementary file 1 [file DataSheet_1.docx]
