# Supplementary material for: Comprehensive analysis of LRR-RLKs and key gene identification in Pinus massoniana resistant to pine wood nematode
Source: Front Plant Sci. 2022 Dec 14;13:1043261. doi: 10.3389/fpls.2022.1043261 (PMC9795191; doi:10.3389/fpls.2022.1043261)
Supplement: Supplementary file 2 [file Table_1.docx]

**Table S1. Quantitative primers.**

|  |  |  |
| --- | --- | --- |
| Genes name | Position | GSP sequence（5’-3’） |
| PmRLKs1 | F | CATCACTCGTGTCGCCAAAG |
|  | R | GGCCTGGAAGACGGTTCAG |
| PmRLKs4 | F | TGCTGGGCTCTGTGTTTCC |
|  | R | TGGGTTGAATGATGGGTTTCA |
| PmRLKs13 | F | AAATTCTTAATGTCTCGTCAAACTCACT |
|  | R | GATCCATAATTGTGCAGCTTCCT |
| PmRLKs23 | F | CTCCAACTGTCGTGATTTAGTCCTT |
|  | R | TGATGAGGAATTTGGCCAATG |
| PmRLKs31 | F | ATCAGCCAGACCAACAACAGAA |
|  | R | TCCAGGCCTTGACCACAGA |
| PmRLKs32 | F | GTGACTCCCAGCGCAGATGT |
|  | R | CTTTTCCTCGTCAATAATTCCATGA |
| PmRLKs34 | F | TGGCTTCCTTAGGGTCTCGTT |
|  | R | TGCCGCGTCGGTTTG |
| PmRLKs36 | F | CAGGAGGCATTGTGAATTCCA |
|  | R | GGAGTCGAGGGCACAATACTTG |
| PmRLKs39 | F | TCGCGACTGCAGCTGTATTC |
|  | R | TGCGCACTTGACACTTTTATCA |
| PmRLKs65 | F | GACCATCGATGAGGGAAGTAGTG |
|  | R | GAAGCCTGAACCCATTTCCA |
| PmRLKs73 | F | CTGGACGCCTCGCAAAAT |
|  | R | TCCTCCTTTGCAGATATCTTTGG |
| PmRLKs93 | F | TGATTGGGAGCGGAGCAT |
|  | R | TGCTACAGTTTCATTGTTGGGTATG |
| PmRLKs94 | F | GCGTACTGCTGCTCTTTCAATTT |
|  | R | GGCGGGATTGCACATCTC |
| PmRLKs106 | F | CGGCTTGCTCGACTTCTTCTTAT |
|  | R | TTAGACCGGCAGCCAATTTC |
| PmRLKs124 | F | CCTTTATCTGTTTGGGCACTTGA |
|  | R | GCACCCACGTCCAAGGAA |
| PmRLKs141 | F | GCAGAGTCTCAGCCGGAAAC |
|  | R | AGAGCTCCCCTCCCTTGAAA |
| PmRLKs147 | F | TGCCCAATGGCGAGGTAA |
|  | R | CGATTGGTTTCTTTCTGGTTTTTC |
| PmRLKs148 | F | GCCGTTCTCTTGTCTGTTTTCA |
|  | R | TTGCGGAGGGCAGATGAT |
| PmRLKs153 | F | TGGCTCTGTCCCTGTTGGTTA |
|  | R | GGCCAGGATTGCGAAGAA |
| PmRLKs159 | F | GCTTTATGGGCAATGTAGCACTTT |
|  | R | TCCTGTCCTGGAGCAATGTTT |
| PmRLKs174 | F | GTGTCCACGGAGTTCGGTAAA |
|  | R | GCTGCCGCTAACCAGTTGA |
| PmRLKs181 | F | GTTGCAGGTTCCTTTGGATACAT |
|  | R | AGCTGCTGTCACTTGCATGGT |
| PmMMK1 | F | CTGAGGCTGACCTTGGATTTG |
|  | R | TGGCAGTTGCCGAATAAATCT |
| PmMPK9 | F | CCAGGTTATTAAGGCAAATGATGA |
|  | R | GTAGAAGCTGGTACAGGAAAAATTGA |
| PmMPK | F | ACGAGGAGCATACGGTGTGAT |
|  | R | GCAACTTGCTCATTGGTTTCTG |
| PmSERK1 | F | TGGTGGCGTCGCAGAAA |
|  | R | GGATCCTCCTCAGCAGGTACAT |
|  |  |  |
